# The use of a geostatistical model supported by multivariate analysis to assess the spatial distribution of mercury in soils from historical mining areas: Karczówka Mt., Miedzianka Mt., and Rudki (south-central Poland)

**DOI:** 10.1007/s10661-019-7368-5

**Published:** 2019-04-24

**Authors:** Sabina Dołęgowska, Artur Michalik

**Affiliations:** Geochemistry and the Environment Division, Institute of Chemistry, Jan Kochanowski University, 15G Świętokrzyska St., 25-406 Kielce, Poland

**Keywords:** Mercury, Post-mining areas, GIS, Geological factors, Factor analysis, Cluster analysis

## Abstract

For the purpose of this study, 181 soil samples were collected from three post-mining areas (Miedzianka Mt. (62), Karczówka Mt. (61), and Rudki (58)) in the Holy Cross Mountains, south-central Poland. Collected samples were dried, disaggregated, and digested in a closed microwave system. All solutions were analyzed for Hg concentrations with cold vapor-atomic absorption spectroscopy (CV-AAS) technique using a continuous flow vapor accessory. The average Hg concentrations and the upper limits of geochemical background (UBG) were as follows: Miedzianka Mt. Hg 0.501 mg kg^−1^, UBG 0.312 mg kg^−1^; Karczówka Mt. Hg 0.150 mg kg^−1^, UBG 0.180 mg kg^−1^; Rudki area Hg 0.216 mg kg^−1^, UBG 0.193 mg kg^−1^. The use of a spatial distribution map of mercury concentrations integrated with computed geochemical factors and results of cluster analysis showed a direct relationship between mercury contents and mining activity conducted in these areas. Only in the case of Miedzianka Mt., this relationship was visible and probably resulted from the presence of tennantite (Cu,Fe)_12_As_4_S_13_ in soil samples, which was also confirmed with the factor analysis. Higher Hg concentrations in soil samples from Karczówka Mt. and Rudki resulted from the presence of clay and other secondary minerals that increase the mercury adsorption from atmospheric deposition. Fossil fuel and biomass combustion was classified as the main anthropogenic source of the metal, but the neighborhood of a cement factory may be taken under consideration. Our results showed that the use of integrated geostatistical models allows for better data visualization and interpretation.

**Electronic supplementary material:**

The online version of this article (10.1007/s10661-019-7368-5) contains supplementary material, which is available to authorized users.

## Introduction

Abandoned mining areas belong to the most dangerous uncontrolled sources of pollutants (Kim and Hyun 2015). For several years, these areas have gained significant attention to scientific community (Bori et al. 2016; Bosso and Enzweiler 2008; Castillo et al. 2013; Gałuszka et al. 2016; Sheoran et al. 2010; Xu et al. 2017). This is not surprising, given the fact that many of them have not been submitted to the recovery program. The degree of contamination of post-mining areas varies and depends on several natural and anthropogenic factors, such as weathering of mineralized zones and/or mining operations and processes, like smelting or waste disposal. For example, degradation of soils leads to changes in pH, EC, and trace element concentrations and subsequently to formation of technosols classified as soils that strongly influence human materials (Kim and Hyun 2015; Uzarowicz 2011).

Previous studies of abandoned mining areas carried out in the Holy Cross Mountains (HCM) (Gałuszka et al. 2018, 2016, 2015; Migaszewski et al. 2015; Uzarowicz 2011) reported high levels of some trace elements in examined soils and their high heterogeneity (Dołęgowska et al. 2015). As documented by Gałuszka et al. (2016, 2015), most of the elements examined (e.g., As, Cd, Cu, Pb, and U) showed higher mean contents than recommended standard values for soils in protected areas of Poland, but concurrently, they were lower than those noted in soils of industrial areas. Of the wide range of elements determined, no studies have been conducted on mercury which is regarded as a highly toxic element, and its concentrations in surface soil horizons can be a potential threat to terrestrial organisms.

Mercury occurs in the environment as a relatively stable metallic element (Hg^0^) and as mono- and divalent ions. Divalent ions are more common in the environment and in the particulate phase due to their greater stability (Carpi 1997; Schroeder and Munthe 1998). Chemical behavior of mercury in soils depends on several parameters which may change its chemical species and mobility. For example, the presence of clay minerals, Mn and Fe oxides and hydroxides, and organic matter favors adsorption processes of mercury whereas the occurrence of chloride ions reduces sorption and enhances the complexation processes (Bonnissel-Gissinger et al. 1999; Jackson 1989; Kongchum et al. 2011). Kabata-Pendias (2010) reports that the mean mercury concentrations in surface soils usually do not exceed 0.400 mg kg^−1^ whereas normal soils contain about 0.020–0.150 mg kg^−1^ (Geological Survey of Finland 2001). Mercury is emitted into the atmosphere from natural (e.g., volcanic eruptions, wild fires, and geothermal activity) and anthropogenic sources (e.g., mining operations, coal and fossil fuel combustion, cement production, and waste disposal). The correct discrimination between natural and anthropogenic sources of mercury in soils is crucial for proper assessment of the soil contamination level. The heterogeneous character of historic metalliferous ore mining areas makes the evaluation of element provenience far more problematic due to the human-induced geochemical processes that changed the previous natural conditions.

So far, several geochemical factors, such as geochemical background (BG), enrichment factor (EF), or geoaccumulation index (*I*_geo_), have been used to determine the origin of elements and their pollution levels (Barbieri 2016; Hani et al. 2010; Martínez et al. 2007; Tume et al. 2017). These simple tools may indicate “normal” and “hot spot” concentrations and facilitate the comparison of enrichment points with different environmental aspects (Barbieri 2016). However, as shown by Desaules (2012), there is no one precise and accurate method for assessing the soil contamination level. All the factors mentioned above can be more useful when combining with other multivariate statistical techniques, such as cluster and factor analyses and geographical information system (GIS). In practice, the use of integrated geostatistical tools gives a better understanding of the spatial distribution of elements and relationships between different environmental factors.

The main objectives of this study were to (i) visualize and characterize the spatial distribution of Hg in soil samples collected within three post-mining areas and (ii) identify and discuss different mercury pollution sources using a combination of selected geochemical factors and statistical analyses with a GIS spatial analysis model.

## Materials and methods

### Study areas—short geological characterization

**Fig. 1:**
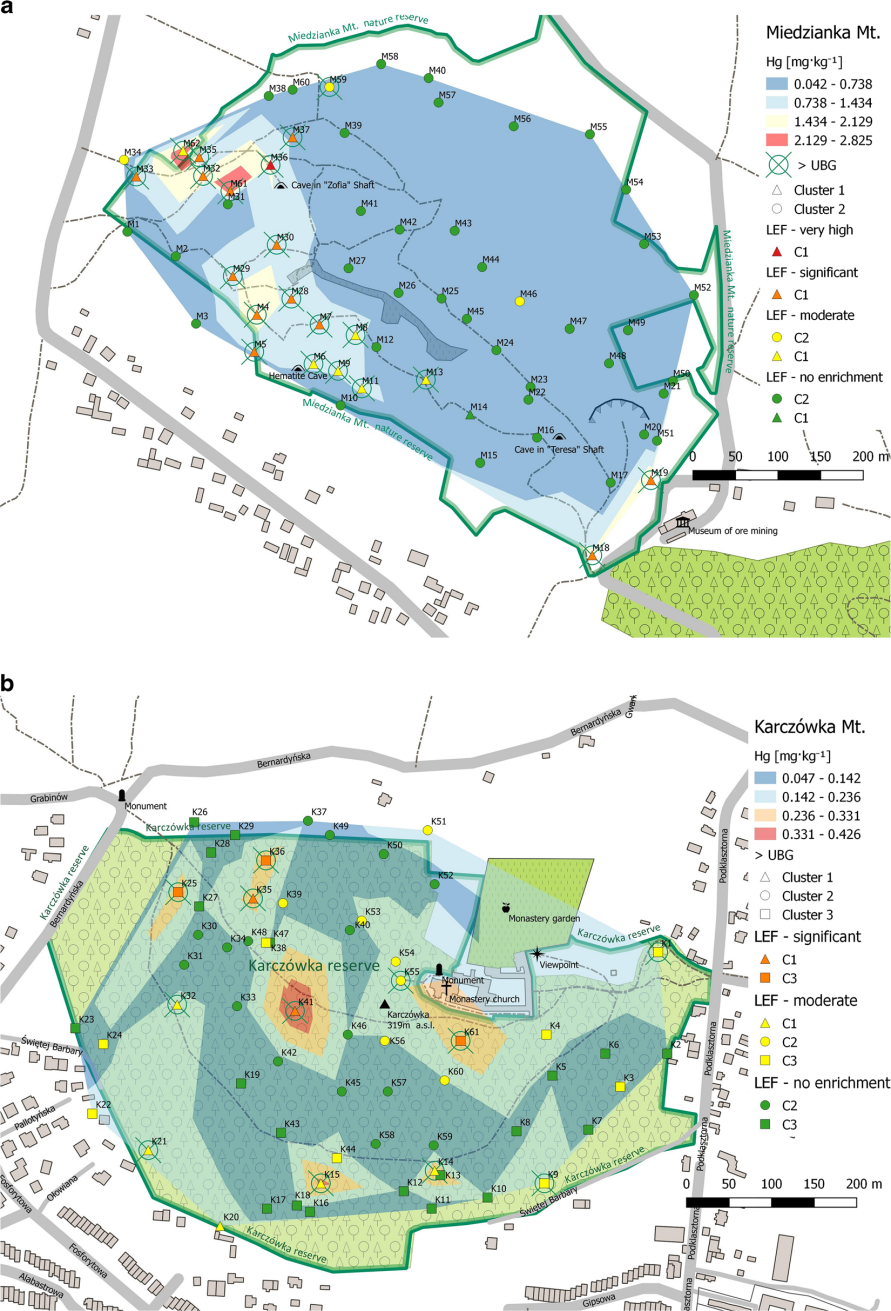

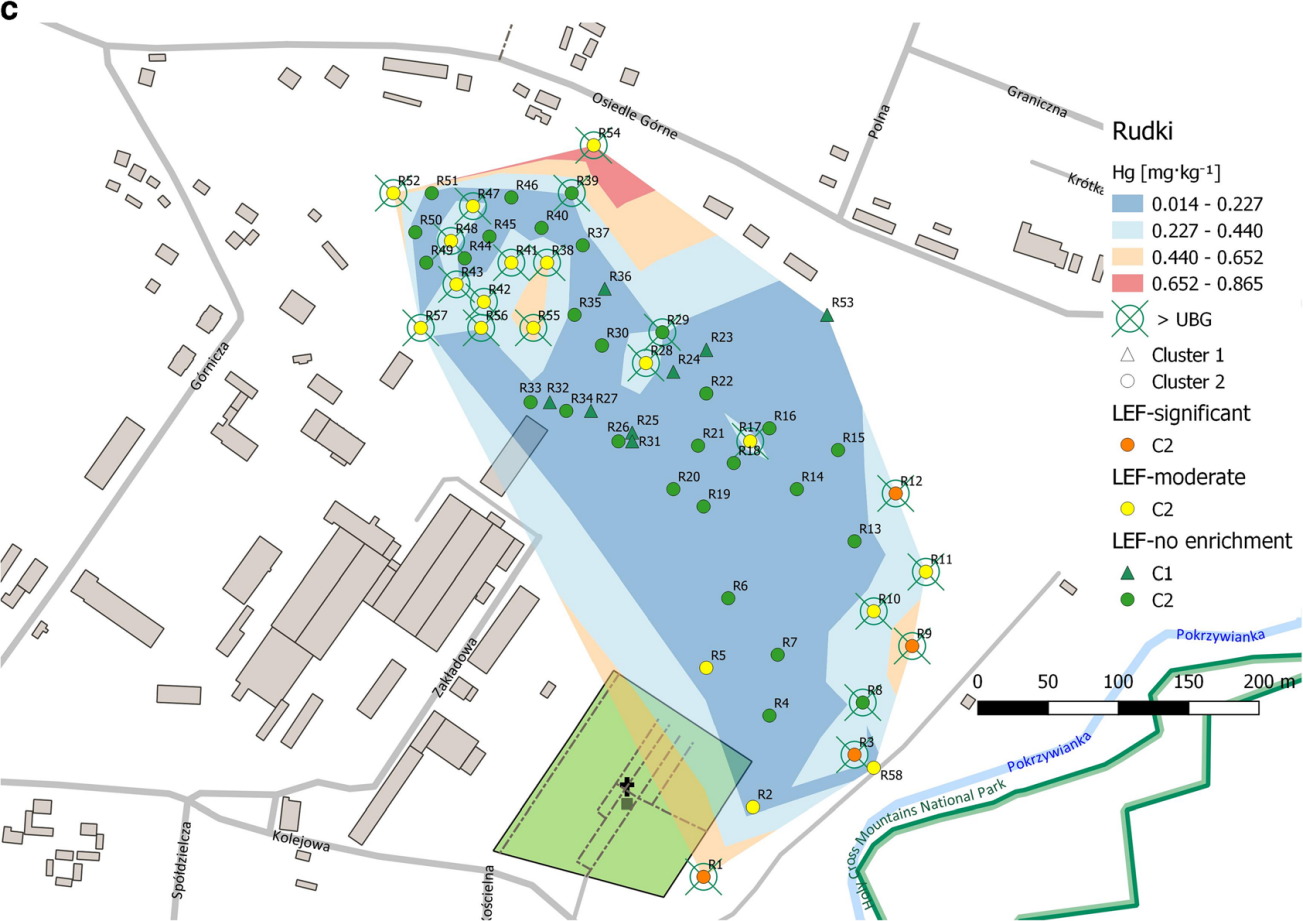
Spatial distribution map of mercury integrated with computed geochemical factors and cluster analysis. **a** Miedzianka Mt. **b** Karczówka Mt. **c** Rudki

Localization of examined areas is presented in Fig. 1 of ESM 2. The study was conducted within three historical mining areas: Miedzianka Mt., Karczówka Mt., and Rudki. Their detailed characterization is compiled in Table 1.

**Table 1:** Short characterization and geological description of study areas

	Miedzianka Mt.	Karczówka Mt	Rudki area
Localization	Southwestern part of the parallel Chęciny Anticline (Gałuszka et al. 2015)	Western part of the Kielce city, the Kadzielnia-Białogon Range (Migaszewski et al. 2015)	The Bodzentyn syncline, north-central part of the Holy Cross Mountains (Nieć 1969)
Mines by type	Copper mine	Lead mine	Pyrite-uranium mine
Mining activity	From early Middle Ages until 1953	From late Middle Ages until the First World War	From the Roman period until 1971
Lithology	1. Middle and Upper Devonian limestones pierced with calcite and copper sulphfide veins or with calcareous-clayey shale lenses and interbeds 2. Lower Cambrian mudstones and sandstones 3. Pleistocene fluvioglacial sands with subordinate tills covering rock formations (Swęd et al. 2015)	1. Middle and Upper Devonian limestones, faulted and fractured, forming a system of veins filled mainly with calcite and galena ore (Urban and Gagol 2008; Zieliński et al. 2016)	1. Silurian clayey-silty shales and sandstones 2. Lower and Middle Devonian carbonate rocks
Mineral composition	1. Primary sulfide deposits: chalcopyrite, covellite, chalcocite, and tennantite	1. Calcite veins (associated with Variscan orogeny)	1. Pyrite-hematite-siderite-uranium mineral deposit linked to the presence of the deep-rooted Łysogóry fault
	2. Secondary sulfide deposits associated with a secondary mineralization: cuprite, tenorite, malachite, azurite, conichalcite, cornubite, olivenite, zincian olivenite, tyrolite, hentschelite, pseudomalachite, antlerite, brochantite, and marshite	2. Calcite veins enriched in galena, pyrite, marcasite, barite, chalcopyrite, and sphalerite (associated with Alpine orogeny)	2. Claystones with limonite and hematite (weathering products) (Gałuszka et al. 2016)
Works after mine closure	1953: shutdown of mining operations; lack of reclamation works	1971: shutdown of mining operations; lack of reclamation	1971: shutdown of mining operations and reclamation of the post-mining area; dumping of mining waste in the neighboring Serwis village
	1958: establishment of a nature preserve	1953: establishment of a nature preserve	
Soil characterization and land use	Leptosols, cambiosols, and technosols	Sandy rendzinas, arenosols, and lithosols	Spolic technosols (Uzarowicz 2011)
	Nature preserve	Nature preserve	Sparsely vegetated area with muddy patches, not used since the reclamation process

### Sample collection and preparation

Soil samples were collected in November of 2012 (Miedzianka Mt. and Karczówka Mt.) and in May of 2013 (Rudki). Locations of sampling points are presented in Fig. 1a–c. Soil samples from Miedzianka (62), Karczówka (61), and Rudki (58) were collected using a systematic random strategy. Samples (about 2 kg each) were taken from a depth interval of 0.2–0.5 m. Each sample consisted of 5 to 10 increments (subsamples) collected within an area of about 1 m^2^. Just after sampling, soil increments were cleaned from alien materials and oversized particles (*Ø* > 2 mm) and homogenized using a cone and quarter technique. In addition, duplicates of eight samples from Karczówka and ten samples from Miedzianka were collected using the same sampling procedure. The preliminary prepared samples were placed in signed ziplock polyethylene bags and transported to the laboratory.

At the laboratory, soil samples were dried at an ambient temperature of about 20 °C and then disaggregated to pass a 0.063-mm sieve using a Pulverisette 2 Fritsch grinder and an Analysette 3 Spartan shaker (FRITSCH, Germany). Before analysis, the soil samples (0.5 g each) were digested with aqua regia (6 mL of 30% HCl Suprapur® and 2 mL of 65% HNO_3_ Suprapur^®^) in a closed microwave system Multiwave 3000 (Anton Paar, Austria). After digestion, the samples were replenished up to 25 mL with deionized water and filtered to disposable Falcon tubes. To reduce the loss of mercury during sample preparation and analysis, adequate safety precautions were taken. The digestion procedure was provided according to the parameters presented in Table 1 of ESM 1. All reagents were free of mercury whereas the glassware was prepared by washing with detergent and rinsing and soaking in 5% HNO_3_ for 1 week. Subsequently, it was rinsed with deionized water and dried. Just before analysis, the absorption cell and silicone tubing were warmed to avoid the condensation of water vapor during the analysis.

### Chemical analysis

For the purpose of this study, the digested solutions were analyzed with cold vapor-atomic absorption spectroscopy (CV-AAS) technique. In this technique, mercury is reduced to the metal form (Hg^0^) and then transferred to the vapor phase. The possibility of using this technique is related to the specific properties of mercury which exhibits very high vapor pressure (0.0016 mbar) under an ambient temperature (20 °C). This property allows us to analyze mercury using the AAS method but without an atomization step. The most problematic are interferences associated with the condensation of water vapor in the silicone tubing and absorption cell. However, this inconvenience can be successfully reduced by warming the previously mentioned accessories (Welz and Sperling 2007).

The samples, 5% (*v*/*v*) hydrochloric acid and 5% (*m*/*v*) sodium tetrahydroborate (II) stabilized in 0.5% (*m*/*v*) sodium hydroxide, were pumped using a VP100 continuous flow vapor accessory to the reaction zone, mixed, and transported to a gas-liquid separator. All reagents were prepared immediately before analysis. Mercury ions were reduced to the metal species and transferred in a gaseous form to the absorption cell. The gas-liquid separator was equipped with a semipermeable Teflon membrane which prevented from carrying moisture and salts over into the measurement cell. The flow rates of the sample, acid, and reductant channels were 7.5 mL min^−1^, 0.7 mL min^−1^, and 1.6 mL min^−1^, respectively. Parameters of the AAS instrument are summarized in Table 1 of ESM 1. For the quality control of the analysis, the recalibration process was done after a series of ten samples analyzed and certified reference material GSS 4 (Chinese Academy of Geological Sciences) was used. The calculated limits of determination and quantification were as follows: 0.182 mg L^−1^ and 0.546 mg L^−1^, and the average percentage recovery was 109%. The RSD values were < 5% for all the samples analyzed.

### Characteristics of geochemical factors, statistical analyses, and data mapping

Statistical analysis of data was done with the TIBCO^®^ Statistica™ software, while spatial visualization was carried out using the Quantum GIS Mapping Tool program (QGIS Development Team 2017).

#### Geochemical background

A factor used to distinguish natural and anthropogenic concentrations of elements in the environment (Matschullat et al. 2000). Its main goal is to eliminate from datasets all extreme values that are related to anthropogenic sources and whose presence in the datasets disturbs the natural scatter of the background. For the purpose of this study, the geochemical background was calculated using the iterative 2*σ*-technique (Gałuszka and Migaszewski 2011). In this technique, background concentrations correspond to the values within the range mean ± 2*σ*.

#### Enrichment factor

A factor practically used for assessing the origin of elements in the environment and for determining the amount of element introduced from anthropogenic sources. This normalizes a metal content against a reference element, conservative and not anthropogenically altered, like Al, Sc, or Ti (Ghrefat et al. 2011; Yongming et al. 2006). In this study, the modified local enrichment factor (LEF) was computed employing the following equation:

$$ {\mathrm{LEF}}_{\mathrm{e}\mathrm{lement}}=\frac{A_{\mathrm{e}}\times {B}_{\mathrm{BG}}}{A_{\mathrm{BG}}\times {B}_{\mathrm{e}}} $$

where *A*_e_ and *B*_e_ are the element and reference element concentrations in an environmental sample, respectively, and *A*_BG_ and *B*_BG_ are the upper limits of geochemical background range of the element and the reference element, respectively.

The use of the geochemical background of the element instead of the Clarke values enables us to consider local geochemistry (Gałuszka et al. 2016). The computed geochemical background and local enrichment factor values are compiled in Table 2. To be consistent with our previous work (Gałuszka et al. 2015), Ti was used as a reference element. For this element, the average values were as follows: 52.0 mg kg^−1^ for Miedzianka Mt., 8.77 mg kg^−1^ for Karczówka Mt., and 540 mg kg^−1^ for Rudki area.

**Table 2:** Summary statistics (range, mean, and median), geochemical background (BG), and local enrichment factor (LEF)

Parameter		Miedzianka Mt.	Karczówka Mt.	Rudki
Concentration (mg kg^−1^)	Range	0.040–2.825	0.05–0.430	0.04–0.860
	Mean	0.501	0.150	0.216
	Median	0.210	0.130	0.156
BG (mg kg^−1^)	Mean	0.160	0.120	0.110
	Upper limit of BG	0.312	0.180	0.193
LEF		Sampling sites		
No enrichment or minimal enrichment	< 2	M1–3, M10, M12, M14–17, M20–27, M31, M38–45, M47–58, M60	K2, K5–8, K10–13, K16–19, K23, K26–31, K33, K34, K37, K40, K42, K43, K45–50, K52, K57–59	R4, R6–8, R13–16, R18–27, R29–37, R39, R40, R44–46, R49–51, R53
Moderate enrichment	2 ≤ EF < 5	M6, M8, M9, M11, M13, M34, M46, M59	K1, K3, K4, K9, K14, K15, K20–22, K24, K32, K38, K39, K44, K51, K53–56, K60	R2, R5, R10, R11, R17, R28, R38, R41–43, R47, R48, R52, R54–58
Significant enrichment	5 ≤ EF < 20	M4, M5, M7, M18, M19, M28–30, M32, M33, M35, M37, M61	K25, K35, K36, K41, K61	R1, R3, R9, R12
Very high enrichment	20 ≤ EF < 40	M36	–	–
Extremely high enrichment	≥ 40	–	–	–

#### Cluster analysis (CA)

A type of multivariate analysis successfully used to group and classify objects on the basis of the characteristics they have. The clusters of objects are a graphical presentation of similarities that were found between data by reduction in dimensionality of the original dataset. They have high external heterogeneity and high internal homogeneity. For a long time, cluster analysis has been used as a simple statistical tool for visualization of similarities in the entire dataset (Dragović and Mihailović 2009; Martínez et al. 2007; Yongming et al. 2006).

For the purpose of this study and for a better understanding of mercury provenience, the cluster analysis was done for non-mercury-biased datasets and the results were presented as one of the thematic layers on the spatial distribution map of mercury. Due to the high positive skewness of analyzed data, they were normalized using the Box-Cox function before the proper analysis was done. Subsequently, the cluster analysis was performed using Ward’s method, and 1-Pearson *r* distances as a measure of similarity. The 1-Pearson *r* distance is defined as *d*_*ij*_ = (1 − *r*_*ij*_)/2, where *d*_*ij*_ is the distance between samples *i* and *j* and *r*_*ij*_ is the Pearson correlation coefficient between samples *i* and *j*. For similar samples, the correlation coefficient is close to one and the distance between the samples is close to zero. The distances between clusters were evaluated with Ward’s method (Ward’s minimum variance method) which is based on the analysis of variance. The spatial variability of chemical characteristics of soils in all sampling points was determined using the linkage distance and reported as *D*_link_/*D*_max_, which represents the quotient between the linkage distances for a particular soil sample divided by the maximal linkage distance (Everitt et al. 2011). The results of the cluster analysis (CA) are presented in Fig. 2a–c of ESM 2.

#### Factor analysis (FA)

A type of multivariate analysis based on the correlation found between variables and reconstructed by a smaller set of parameters (factors). Factors represent the whole dataset structure in a concise and interpretable form (Basilevsky 2009). In this analysis, each parameter adds dimension to the space which represents our dataset. That is why the measurement of “*n*” parameters requires the use of *n*-dimensional space which represents all these relationships. The final interpretation of the factors is based on the contribution which each original variable has to the linear combination describing the factor axis. This contribution is called the factor loadings and variables with large loadings are identified as associated. In fact, each factor loading indicates how much a factor explains a variable. After reduction, the dimensionality data can be presented in two or three dimensional plots (Breen and Robinson 1985).

For the purpose of this work, factor analysis (FA) was used to find correlations between the content of mercury and other elements examined in the same soil samples and presented in our previous works (Gałuszka et al. 2018, 2016, 2015). Factors were extracted using the PCA (principal component analysis) method. The number of factors was determined using the eigenvalues and Kaiser’s criterion which assume that only factors with values greater than 1 are taken under consideration. Elements grouped in a given factor are correlated and defined by a factor matrix after the previous raw varimax rotation. The raw varimax rotation is performed directly on the loading matrix to increase the factor simplicity (Forina et al. 2005). The results of the FA are presented in Table 2 of ESM 1. The standard for prominent loading was set on 0.70.

#### Geographic information system

A geostatistical tool used to storage and visualize information in combination with geographical data. In this work, the spatial distribution map of Hg was prepared using the Quantum GIS Mapping Tool program. Subsequently, the GIS spatial analysis model was combined with the results of cluster analysis and geochemical factors (BG and LEF) computed. During this operation, four thematic layers were created (Fig. 1a–c). The first layer is a classical contour map of mercury content. This layer presents sampling sites lying between two contour lines which correspond to the maximum and minimum content of Hg specified for each concentration range. At this step, no approximation method was used. In all cases, the same number of concentration ranges was created. However, the upper limits of absolute values depended on the individual Hg concentrations. This type of spatial models enables quick and easy analysis of the results.

The second layer groups all sampling sites where the mercury content exceeded the upper limit of the geochemical background (> UBG) calculated for each study area. The third layer shows information obtained from the cluster analysis. Sampling sites grouped at each cluster are highlighted with a different marker. As mentioned before, the cluster analysis was done for non-mercury-biased datasets. The last layer presents the calculated LEF. Sampling sites are marked with a different color in accordance with the level of enrichment.

## Results and discussion

According to the literature review, the average mercury concentrations in soils vary from 0.05 to 0.3 mg kg^−1^; normal soils contain about 0.020–0.150 mg kg^−1^, while in industrial regions, these concentrations may reach even 40 mg kg^−1^ (Pirrone et al. 2010; Zhu and Zhong 2015). Because atmospheric deposition is one of the most important Hg sources, higher concentrations of this element usually occur in topsoil than in subsoil. In this study, the highest mean Hg concentration was noted in soil samples from Miedzianka Mt. (0.501 mg kg^−1^). As for Karczówka Mt. and Rudki area, the average values were even three times lower: 0.150 mg kg^−1^ and 0.216 mg kg^−1^, respectively. It should be stressed that all these values do not exceed the admissible values specified in Regulation of the Ministry of Environment (2002) for soils in protected areas (0.5 mg kg^−1^) and the regulatory reference values for negligible risk for Hg in soils of the European Community (Desaules 2012). The upper limits of the geochemical background calculated for each area were 0.312 mg kg^−1^ for Miedzianka Mt., 0.180 mg kg^−1^ for Karczówka Mt., and 0.193 mg kg^−1^ for Rudki area. The computed LEF confirmed very high enrichment in Hg (LEF > 20) only in one sampling point (M36) from Miedzianka Mt. whereas a significant enrichment was noted in 12 of 62 samples. In most samples from Karczówka Mt. and Rudki, moderate and very low enrichments were reported. Summary statistics (mean, median, range, and standard deviation) and geochemical factors (BG, LEF) are compiled in Table 2.

Optical microscope studies and electron microprobe analysis of selected soil samples showed that soil samples from Miedzianka Mt. and Rudki area are enriched in clay minerals, iron/manganese oxides and hydroxides, and different copper and iron minerals. However, no Hg minerals were recorded (Gałuszka et al. 2016, 2015) (Fig. 1).

### Miedzianka Mt.

The average content of mercury in soil samples from Miedzianka Mt. (0.501 mg kg^−1^) was slightly higher than the mean concentrations found in most surface soils (0.400 mg kg^−1^). This suggests that even in the absence of specific mercury minerals in soil samples, higher concentrations of this element (Table 2) may be linked with mineralogy of the study area (Table 1). One of the copper mineral found in soils from Miedzianka was tennantite, a primary Cu mineral that belongs to the tetrahedrite group of minerals (Gałuszka et al. 2015). According to the literature, this mineral can hold significant amounts of several trace elements, such as As, Hg, and Sb. Mercury contents in tennantite may reach even 400 mg kg^−1^ (Bautista 2013; Ridge 1984). High values of Spearman’s correlation coefficient computed for the following pairs of elements Hg-Sb (0.84) and Hg-As (0.83) seem to confirm this hypothesis (results for the other metals were taken from Gałuszka et al. 2018, 2016, 2015). Moreover, the statistically significant correlation was also noted between Cu and Sb (0.94), As and Sb (0.93), Cu and As (0.89), and Cu and Hg (0.85). A significant enrichment of samples in As reported by Gałuszka et al. (2015) suggests the presence of As-rich tennantite. However, the occurrence of mercury as a result of specific substitution in the crystal structure is also possible. During the factor analysis, three main factors accounted for 79% of the total variability were extracted (Table 2 of ESM 1). Hg was loaded on factor 2 along with As, Ag, Sb, Pb, and Bi. This may be indicative of the same geogenic origin of Hg, Sb, and As. Interestingly, the statistically significant correlation was also found between Hg content and pH of soil water extracts (0.57). The pH of soils was neutral or close to neutral (5.6–7.3) (Gałuszka et al. 2015). As it was shown by Yang et al. (2007), acidic conditions may increase mercury bioavailability and migration; however, this relation cannot be discussed without the characterization of soil properties and other interacting factors of environmental parameters (e.g., organic matter content). Hg dissolution decreases in the range of pH 7.0–9.0 (Xu et al. 2014). In our studies, the highest Hg concentrations were noted in samples with pH in the range from 6.6 to 7.3.

Copper mining is listed as a one of the major sources of Hg (Pacyna et al. 2007, 2009; Pacyna and Pacyna 2002; Pirrone et al. 2010; Streets et al. 2011). The spatial distribution map of mercury combined with computed geochemical factors (BG, LEF) and CA results (Fig. 1a) indicates a direct relationship between the mercury content and mining operations. The distribution of mercury has a boundary in the southeastern part of the study area, which is also less wooded and more exposed. In most cases, the concentration of Hg in soil samples taken from this part exceeds the upper limit of the geochemical background value (0.321 mg kg^−1^) (Fig. 1a). The computed LEF confirmed moderate (2 ≤ LEF < 5) and significant (5 ≤ LEF < 20) enrichment in mercury. A very high enrichment (20 ≤ LEF < 40) is observed only in sample M36. Practically, all the sampling sites located in this part are grouped in cluster 1 (Fig. 2a of ESM 2). Based on the information about the historic mining activity, most of these sites are located in the immediate vicinity of abandoned mine waste heaps, pits, and shafts (Gałuszka et al. 2015). What is more, soils from this part are represented mainly by technosols.

Higher concentrations of metals including mercury can be also found in secondary clay minerals (kaolinite and illite) and in organic matter. These two factors regulate metal-solid interactions and make adsorption of mercury more efficient. The presence of clay minerals enhances the retention of mercury (Kongchum et al. 2011; Volzone and Hipedinger 1997). Optical microscope studies and electron microprobe analysis showed that the samples from Miedzianka Mt. are mostly a mixture of clay minerals, quartz, subordinate calcite, muscovite, and feldspars (Gałuszka et al. 2015). Bulk soils (< 2 mm) and clay fractions (< 0.002 mm) of the samples examined were enriched in kaolinite, illite, and quartz. Selected soil samples grouped in the first cluster (M5, M7, M19, M28, M35, and M62) and enriched in Hg had the bulk fraction dominant and moderately enriched in kaolinite and quartz whereas the clay fraction is dominant and moderately enriched in kaolinite and/or illite and quartz (Fig. 1a).

These results are consistent with the results of a study carried out by Kongchum et al. (2011) who showed that higher concentrations of Hg can be related with the presence of clay minerals whose sorption capacity for this element may reach even 1.3 mg g^−1^ (Sarkar et al. 2000; Zhu et al. 2012). Sorption properties of kaolinite are additionally enhanced by the aggregation of particles which are usually negatively charged. This process is increased by the presence of Ca, Cu, Ni, and Pb. Because all these elements are abundant in the soil samples examined (Gałuszka et al. 2016, 2015), the mercury adsorption from the atmospheric deposition should be also enhanced. In contrast, samples M52 and M53 had a completely different mineralogical composition. In their bulk and clay fractions, quartz was a dominant mineral, while kaolinite and illite occurred in a small amount or did not occur at all. In the clay fraction of M53, only feldspar was in moderate amounts (Gałuszka et al. 2015). In our study, both these sites belong to the second cluster and have much lower mercury contents, 0.067 and 0.065 mg kg^−1^, respectively.

Higher concentrations noted in samples from the south and southwestern parts of the study area can be also linked with the vicinity of single-family houses. We know that mercury is released to the atmosphere from a number of industrial processes among which coal combustion is one of the major pollution sources (Pacyna and Pacyna 2002; Pirrone et al. 2010). Surface soils collected from various areas located in the vicinity of power plants and residential and commercial objects have elevated level of mercury (Fitzgerald and Lamborg 2013). This enrichment is related to the atmospheric deposition of Hg, which is enhanced by the presence of clay minerals and Mn/Fe oxyhydroxides. In accordance with the data obtained by Gałuszka et al. (2015), Mn/Fe oxides and hydroxides also occur in Miedzianka soil samples. Nevertheless, the direct exposure of the southwestern part of the slope to the prevailing southwest wind should also be considered. A cement plant which is located about 10 km to the southwest can be another pollution source of Hg. As shown by different authors (Munteanu and Munteanu 2007; Pacyna and Pacyna 2002; Pirrone et al. 2010; Zhang and Wong 2007), cement industry is a significant source of mercury. Interestingly, lower Hg concentrations were noted in samples M1–M3 located in the southwestern wooded part of the study area. These are grouped in the second cluster and the local enrichment factor indicates no enrichment in Hg (Fig. 1a).

A significant enrichment in Hg (concentrations above the upper limit of the geochemical background; 5 ≤ LEF < 20) was also found on sites M18 and M19. These two sites belong to the first cluster, but they are located in the southeastern part of the study area (Fig. 1a). Their localization points out to other mercury sources, probably connected with the vicinity of the local road and official buildings. Sampling sites located in the southern, northern, and eastern parts of the study area are grouped in the second cluster (Fig. 1a, Fig. 2a of ESM 2). The LEF confirmed that this part is slightly enriched in Hg or that the enrichment is not observed (LEF < 2). This part of the study area is more wooded with soils classified as leptosols and cambiosols.

### Karczówka Mt.

Considering the results from Karczówka Mt. (Fig. 1b), it can be noted that sampling sites with the highest Hg contents are grouped in the first and third clusters. Despite the lack of mercury minerals, there is a relationship between the Hg content and the mining operation carried out on this area. Sites K14 and K41 are located near historic mining fields and abandoned shafts whereas K15 in the vicinity of mine spoils. Sites K25, K35, and K36 are located in the immediate proximity of small shafts indicating the extent of former exploratory workings (Wróblewski 2014). The Hg content in soil samples from these sites exceeds the upper limit of the geochemical background (0.150 mg kg^−1^) twice or even more. The LEF confirmed a significant (K25, K36, K36, and K41) and moderate (K14, K15) enrichment in mercury (Table 2). There is no statistically significant correlation between Hg and Pb (0.44), which may exclude its geogenic origin. It is hard to discuss the content of Hg in different metalliferous ores. According to Streets et al. (2011), all metalliferous ores (Cu, Pb, and Fe) also contain Hg being one of its sources (Pacyna and Pacyna 2002). Mercury can appear in these ores as impurity or as a trace element. For lead ores, this concentration is very low and usually do not exceed 0.500 mg kg^−1^ (Zhang and Wong 2007).

The factor analysis allowed us to extract four major factors, accounting 78% of the total variability (Table 2 of ESM 1). Mercury is classified in factor 2, which is also highly loaded on Pb, Cd, Ag, and As. Of these elements, mercury was slightly correlated with As (0.75) and Cd (0.74). In fact, we did not find any statistically significant correlation for mercury. The highest Spearman’s correlation coefficient (0.78) was noted for the Hg-Zn pair. The same relation, as for samples from Miedzianka Mt., between pH and Hg was noted. The correlation coefficient was 0.63 and was statistically significant. The pH of soils varied from 4.8 to 7.2 (Gałuszka et al. 2018). In most cases, higher Hg contents were in samples with pH higher than 6.4, while lower in samples revealing pH in the range 4.8–5.8.

Interpretation of the results of cluster analysis shows an interesting relationship (Fig. 1b, Fig. 2b of ESM 2). Sampling sites from the central and northwestern parts of the study area are grouped in cluster 2. From this group, sites located in the immediate neighborhood of the Karczówka monastery (K53, K54, K55, K56, and K60) indicate moderate enrichment in Hg, so the monastery and its activity may be considered as one of its major source. The monastery is an important liturgical and missionary center of the region. It is known for the St. Barbara sculpture which is made of one block of galena (Wróblewski 2014). Higher than the upper limit of the geochemical background value is observed only at site K55. Other sites (excluding K33 and K51) which are grouped in cluster 2 do not show an enrichment in Hg. Sampling sites situated in the study area boundary and in its eastern part are grouped in cluster 3. The vicinity of residential buildings and local roads explains higher concentrations of Hg noted at sites K1, K3, K9, K22, and K24 but also at sites K20 and K22 (grouped in cluster 1) and K51 (grouped in cluster 2). At sites K1, K9, and K21, the mercury content is above the upper limit of the geochemical background and the LEF indicates a moderate Hg enrichment. The significant enrichment (5 ≤ LEF < 20) is observed at site K61 located in the vicinity of the Karczówka monastery.

Karczówka Mt. is covered by an old-grown forest with several self-guided trails. According to the literature (Schuster 1991), the air-vegetation exchange is a process occurring in the biogeochemical cycle of Hg. Correlation between the mercury content and the abundance of vegetation is observed in post-mining sites. This may suggest why despite the lack of mercury minerals, sites located in the vicinity of abandoned mining shafts and pits are enriched in this element. It should be also mentioned that the proximity of vegetation may increase the mercury content. However, mercury in plant tissues may be derived mainly from the atmospheric deposition.

Considering this, the main source of mercury in this area is the anthropogenic activity connected mainly with the use of local infrastructures and combustion of fossil fuels (coal, biomass) whereas the former mining activity only induces mercury adsorption (Pacyna and Pacyna 2002; Pirrone et al. 2010). The calculated LEF factor (Fig. 1b; Table 2) confirmed that sandy rendzinas, arenosols, and lithosols of most study areas were not Hg-abundant.

### Rudki

Interpretation of the results from this area is much more problematic because of the reclamation work that was carried out just after mine closure. Mining operations cause changes in natural conditions and in an environmental balance. On one hand, reclamation processes compensate for alternations and make these derelict lands more suitable for further use, but on the other hand, specific conditions remain unchanged. The previous geochemical studies conducted within this area showed soil enrichments in trace elements, such as Pb, As, and U (Gałuszka et al. 2016; Uzarowicz 2011).

There is a weak correlation between the mining activity and Hg content (Fig. 1c). Two major clusters were extracted during cluster analysis (Fig. 2c of ESM 2). The first one, smaller, contains only eight sampling sites located in the central part of the study area (Fig. 1c) and shows no enrichment in Hg whereas the second one contains all the other sites. Interestingly, cluster 2 groups sites with the highest (R1 0.651; R9 0.633; R54 0.865 mg kg^−1^) and also with the lowest (R19 0.041; R20 0.050 mg kg^−1^) mercury content. All the samples in which mercury content was above the upper limit of the geochemical background (0.193 mg kg^−1^) are grouped in cluster 2. Most of them are situated in the western and northwestern parts of the study area. Two are located in the central part and eight in the eastern part (Fig. 1c). These results are in contrary to those obtained for samples from Miedzianka Mt. and Karczówka Mt. where the clusters assigned to mining operations contain only a few sampling sites.

The study of the geological map of the Staszic mine from 1946 (Czarnocki 1947) allowed us to assume that sampling sites from the northwestern part of the study area (R28–R57) are situated near the pyrite and siderite deposits and historic boreholes drilled before 1939 and during 1939–1943 whereas sites R16–R22 are located on the southern side of the historic mine road where some boreholes were also located. The LEF values obtained for these samples confirm a moderate enrichment in Hg in this part of the study area (Fig. 1c; Table 2). Interestingly, a significant enrichment (5 ≤ LEF < 20) is observed only in four samples (R1, R3, R9, and R12) from the eastern part. This may be explained by the location of the former flotation tank (R3–R12), local cemetery, and housing estate (R1).

We found no statistically significant correlations for Hg. The highest Spearman’s correlation coefficients were obtained only for two pairs of elements: Hg-Ag and Hg-Pb (0.74). Optical microscope studies and electron microprobe analysis of selected soil samples from this area confirmed the presence of galena inclusions and veinlets in hematite/goethite and carbonate rocks and a lack of Hg minerals (Gałuszka et al. 2016). Bulk soil samples are composed of quartz and gypsum whereas clay fractions (R5, R14, R22, R33, R48, and R55) are a mixture of illite and kaolinite (Gałuszka et al. 2016). These clay minerals adsorb trace elements, including mercury (Zhu and Zhong 2015). Of this group, only two, R48 and R55, were collected in the vicinity of historic mining sites. In both samples, the content of mercury is above the upper limit of the geochemical background (UBG) (0.193 mg kg^−1^) and the LEF values indicate a moderate enrichment in this element. The pH of soils varied from 2.0 to 7.6 (Gałuszka et al. 2016); however, no relation between those two parameters was observed. The Hg-pH correlation coefficient was 0.14 and was not statistically significant. Interestingly, for samples with the lowest pH values, both high (R55; pH = 3.7, Hg 0.526 mg kg^−1^) and low (R37; pH = 2.0, Hg 0.109 mg kg^−1^) Hg contents were observed.

The results of the factor analysis show that Hg is loaded on the first factor together with Pb, Ag, and Fe. However, this input is not significant (< 0.70) (Table 2 of ESM 1). Due to high heterogeneity of the sampling area, the results of cluster and factor analyses did not give as much information as in the previously discussed areas. The more useful information was obtained from the map of mercury spatial distribution integrated with geochemical factors. As mentioned before, the calculated LEF reports a significant and moderate enrichment in Hg in the western and eastern parts and a minimal enrichment in the central part of the study area (Fig. 1c). These results are consistent with the computed geochemical background. All these results seem to confirm the previous observations that the reclamation was unsuccessful (Gałuszka et al. 2016; Uzarowicz 2011).

The neighborhood of industrial facilities and detached houses located south and southwest of the study area suggests that the anthropogenic activity is the major source of mercury. Correlation observed between the Hg content and historic mining activity results from increased adsorption of atmospheric mercury (from local sources) by altered geologic materials (soils) enhanced by the presence of porous iron oxides and hydroxides (Gałuszka et al. 2016). It should also be stressed that soils of this area are classified as technogenic soils strongly influenced by heterogeneous mines and processing wastes (Uzarowicz 2011).

## Conclusion

Evaluation of metal concentration in soils of post-mining areas is deemed necessary for the assessment of pollution levels and for making further decisions about actions that have to be taken. Heterogeneous and particular characters of these areas make this assessment much more problematic.

Anthropogenic and geogenic sources of mercury have been identified in mine-impacted soils from three post-mining areas using the integrated map of mercury spatial distribution, computed geochemical factors (BG, LEF), and results of the cluster analysis. The use of the combined geostatistical model confirmed a direct relationship between the mercury content and historic mining operations. We documented that despite cessation of mining activity in the middle of the twentieth century and even in the case of Rudki area where the reclamation works have been made, this correlation is still observed. The highest mean mercury concentration was noted in soil samples from Miedzianka Mt. (0.501 mg kg^−1^). A very high enrichment in this metal (20 ≤ LEF < 40) was also reported at one site from this area as a result of Hg-rich copper sulfide occurrences. Due to the lack of mercury minerals in soils from Karczówka Mt. and Rudki, fossil fuel combustion and other emitters (housing estates and local roads) were classified as the major sources of this element. The correlation between the mercury content and historic mining operations can be explained by the presence of clay minerals and Fe/Mn oxides and hydroxides which are scavengers of atmospheric mercury. Results of multivariate analyses carried out for mercury-biased (FA) and non-mercury-biased (CA) datasets emphasized the relation between the presence of other trace metals and mercury.

The use of the integrated geostatistical model which is a combination of multivariate statistics and geostatistical parameters presented by GIS allowed us for an accurate assessment of relationships between the spatial distribution of mercury and other parameters in a small-scale map.

## Electronic supplementary material

ESM 1 (DOCX 25 kb)

ESM 2 (DOCX 884 kb)
