# Supplementary material for: The use of a geostatistical model supported by multivariate analysis to assess the spatial distribution of mercury in soils from historical mining areas: Karczówka Mt., Miedzianka Mt., and Rudki (south-central Poland)
Source: Environ Monit Assess. 2019 Apr 24;191(5):302. doi: 10.1007/s10661-019-7368-5 (PMC6482127; doi:10.1007/s10661-019-7368-5)
Supplement: Supplementary file 1 — (DOCX 25 kb) [file 10661_2019_7368_MOESM1_ESM.docx]

Environmental Monitoring and Assessment, Springer, 2018

Electronic supplementary materials for:

**The use of a geostatistical model supported by multivariate analysis to assess the spatial distribution of mercury in soils from historical mining areas: Karczówka Mt., Miedzianka Mt. and Rudki (south-central Poland)**

Sabina Dołęgowska^a^, Artur Michalik^a*^

^a^Geochemistry and the Environment Division, Institute of Chemistry, Jan Kochanowski University, 15G Świętokrzyska St., 25-406 Kielce, Poland

*Corresponding author: Artur.Michalik@ujk.edu.pl

**Table 1** Parameters of digestion process and AAS instrument.

| **Digestion parameters** | | **Technique** | **Hg** |
| --- | --- | --- | --- |
|  |  |  | **Cold vapor** |
| Sample weight | 500 mg | Type of work | absorption |
|  |  | Type of signal | constant |
| Power | 800 W | Wave length | 253.7 nm |
| Time | 90 min | Lamp current | 75% |
| Time of growth | 15 min | Type of flame | - |
| Time of real digestion | 15 min | Type of buoyant gas | Ar |
| Time of cooling | 60 min | Buoyant gas flow | 100 mL min^-1^ |
| Temperature | 180°C | Background correction | D2 |
| Pressure | 6 MPa | Gap | 0.5 nm |
| p growth rate | 0.03 MPa sec^-1^ | Replicates | 3 |
| Reagents | HCl 6 mL + HNO_3_ 2 mL | Pump speed | 30 mph |
|  |  | Concentration range of standard solutions | 1-20 μg L^-1^ |
|  |  |  |  |
|  | | LOD | 0.182 μg L^-1^ |
|  |  | LOQ | 0.546 μg L^-1^ |
|  |  | Recovery (%) | 109 % |

**Table 2** Rotated factor loadings obtained for study areas.

# Miedzianka Mt.

| **Variable** | **Factor 1** | **Factor 2** | **Factor 3** |
| --- | --- | --- | --- |
| **Ti** | -0,055452 | -0,286741 | 0,706942 |
| **Cr** | 0,332219 | 0,349733 | 0,523278 |
| **Mn** | 0,930296 | 0,079193 | -0,090052 |
| **Fe (%)** | 0,721063 | 0,367658 | 0,422903 |
| **Co** | 0,620444 | 0,621501 | -0,127958 |
| **Ni** | 0,813998 | 0,387204 | 0,300035 |
| **Cu** | 0,741292 | 0,519118 | 0,170126 |
| **Zn** | 0,605238 | 0,438814 | 0,509454 |
| **As** | 0,338412 | 0,876032 | -0,020399 |
| **Ag** | 0,493587 | 0,817607 | 0,090317 |
| **Cd** | 0,745213 | 0,376969 | 0,323348 |
| **Sb** | 0,481159 | 0,794990 | 0,060668 |
| **Ba** | 0,925509 | 0,187793 | -0,083371 |
| **Pb** | -0,128536 | 0,810212 | 0,050901 |
| **Bi** | 0,179291 | 0,701197 | 0,199939 |
| **U** | 0,746537 | 0,307496 | 0,401445 |
| **Hg** | 0,418186 | 0,795994 | 0,108838 |
| **Expl.Var** | 6,244636 | 5,465785 | 1,694751 |
| **Prp.Totl** | 0,367332 | 0,321517 | 0,099691 |

# Karczówka Mt.

| **Variable** | **Factor 1** | **Factor 2** | **Factor 3** | **Factor 4** |
| --- | --- | --- | --- | --- |
| **Ti** | -0,089577 | -0,560387 | -0,247605 | 0,458629 |
| **Cr** | 0,908727 | -0,005086 | -0,138598 | 0,010433 |
| **Mn** | 0,087266 | 0,140572 | 0,727876 | 0,226497 |
| **Fe** | 0,667036 | 0,176759 | 0,622910 | -0,066437 |
| **Co** | 0,927831 | 0,152329 | 0,072073 | 0,104703 |
| **Ni** | 0,951156 | 0,199404 | 0,059795 | 0,024361 |
| **Cu** | 0,744079 | 0,530349 | 0,149102 | -0,038519 |
| **Zn** | 0,582489 | 0,576247 | 0,214389 | 0,026617 |
| **As** | 0,451462 | 0,702411 | 0,202163 | -0,103913 |
| **Ag** | 0,211995 | 0,869892 | -0,060260 | 0,082912 |
| **Cd** | 0,100137 | 0,839330 | -0,091314 | 0,152723 |
| **Sb** | 0,477897 | 0,590291 | -0,337656 | 0,154405 |
| **Ba** | 0,796083 | 0,160300 | 0,430418 | 0,105599 |
| **Pb** | -0,000316 | 0,906446 | -0,102771 | 0,015592 |
| **Bi** | 0,113589 | 0,182521 | 0,077195 | 0,869068 |
| **U** | 0,088891 | -0,339575 | 0,764180 | -0,074288 |
| **Hg** | 0,355634 | 0,816065 | 0,159959 | 0,117627 |
| **Expl.Var** | 5,213076 | 5,006301 | 2,053492 | 1,130779 |
| **Prp.Totl** | 0,306652 | 0,294488 | 0,120794 | 0,066516 |

# Rudki

| **Variable** | **Factor 1** | **Factor 2** | **Factor 3** | **Factor 4** |
| --- | --- | --- | --- | --- |
| **Ti** | -0,404293 | 0,027459 | -0,477782 | -0,113335 |
| **Cr** | 0,302887 | 0,001618 | 0,044715 | 0,869754 |
| **Mn** | 0,110770 | 0,852376 | 0,270360 | 0,073469 |
| **Fe** | 0,703525 | 0,464775 | 0,262957 | 0,093508 |
| **Co** | -0,040692 | 0,371242 | 0,661241 | -0,139087 |
| **Ni** | 0,371602 | 0,671614 | 0,463280 | 0,317573 |
| **Cu** | 0,662891 | -0,337003 | 0,422005 | 0,209179 |
| **Zn** | 0,223747 | 0,151031 | 0,713877 | 0,220565 |
| **As** | 0,347464 | 0,501985 | -0,131298 | 0,388886 |
| **Ag** | 0,889433 | 0,153195 | -0,032971 | 0,234908 |
| **Cd** | 0,130942 | 0,220061 | 0,870103 | 0,064451 |
| **Sb** | 0,113916 | -0,420119 | -0,103410 | 0,202083 |
| **Ba** | 0,174022 | 0,391633 | 0,184587 | 0,806059 |
| **Pb** | 0,874210 | 0,108044 | 0,165621 | 0,305022 |
| **Bi** | 0,568120 | -0,133973 | -0,002850 | 0,636478 |
| **U** | 0,691101 | 0,163968 | 0,399233 | 0,086423 |
| **Hg** | 0,675204 | 0,328923 | -0,000277 | 0,278600 |
| **Expl.Var** | 4,384396 | 2,487045 | 2,719088 | 2,480428 |
| **Prp.Totl** | 0,257906 | 0,146297 | 0,159946 | 0,145908 |

* values in red indicate very strong factor loadings (>0,70).
