# Supplementary material for: The use of a geostatistical model supported by multivariate analysis to assess the spatial distribution of mercury in soils from historical mining areas: Karczówka Mt., Miedzianka Mt., and Rudki (south-central Poland)
Source: Environ Monit Assess. 2019 Apr 24;191(5):302. doi: 10.1007/s10661-019-7368-5 (PMC6482127; doi:10.1007/s10661-019-7368-5)
Supplement: Supplementary file 2 — (DOCX 884 kb) [file 10661_2019_7368_MOESM2_ESM.docx]

Environmental Monitoring and Assessment, Springer, 2018

Electronic supplementary materials for:

**The use of a geostatistical model supported by multivariate analysis to assess the spatial distribution of mercury in soils from historical mining areas: Karczówka Mt., Miedzianka Mt. and Rudki (south-central Poland)**

Sabina Dołęgowska^a^, Artur Michalik^a*^

^a^Geochemistry and the Environment Division, Institute of Chemistry, Jan Kochanowski University, 15G Świętokrzyska St., 25-406 Kielce, Poland

*Corresponding author: Artur.Michalik@ujk.edu.pl


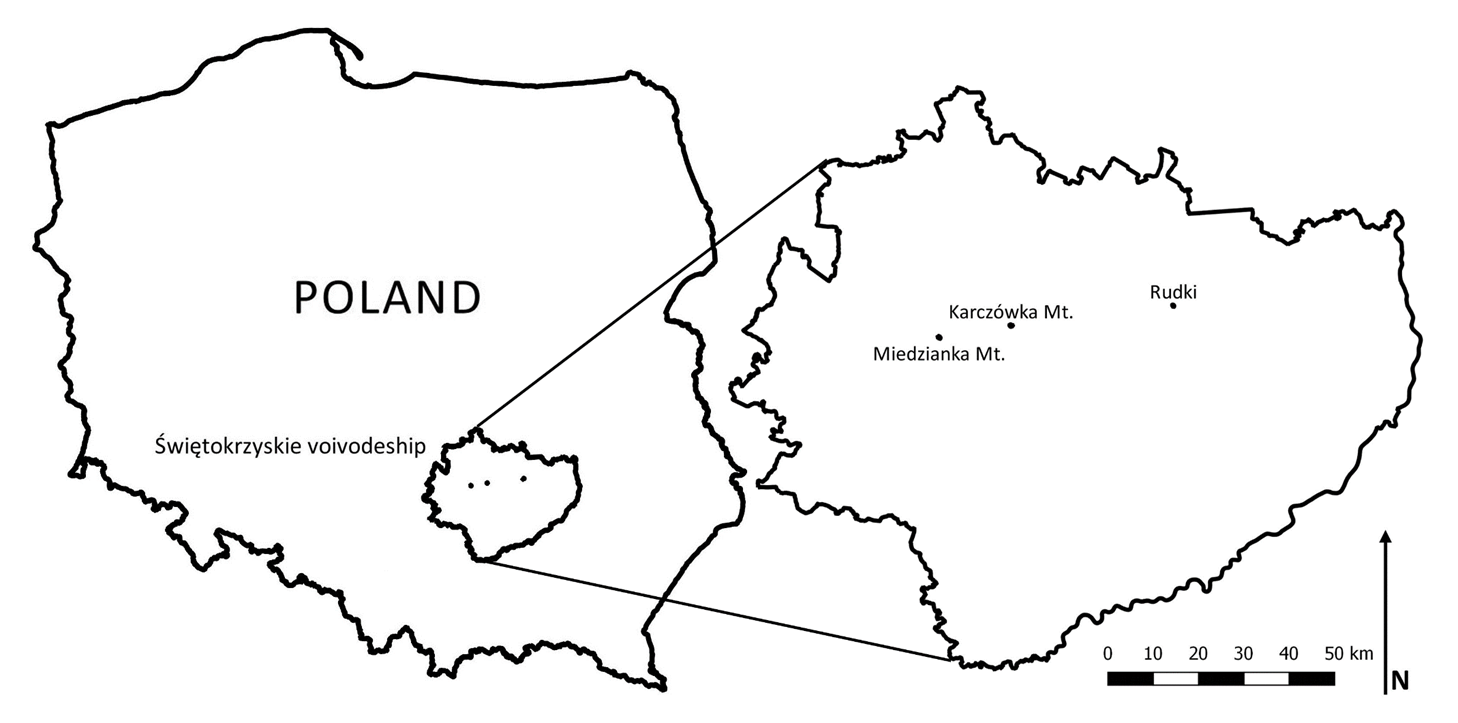


**Fig. 1** Sketch map of the study areas and their localization in Świętokrzyskie voivodeship.


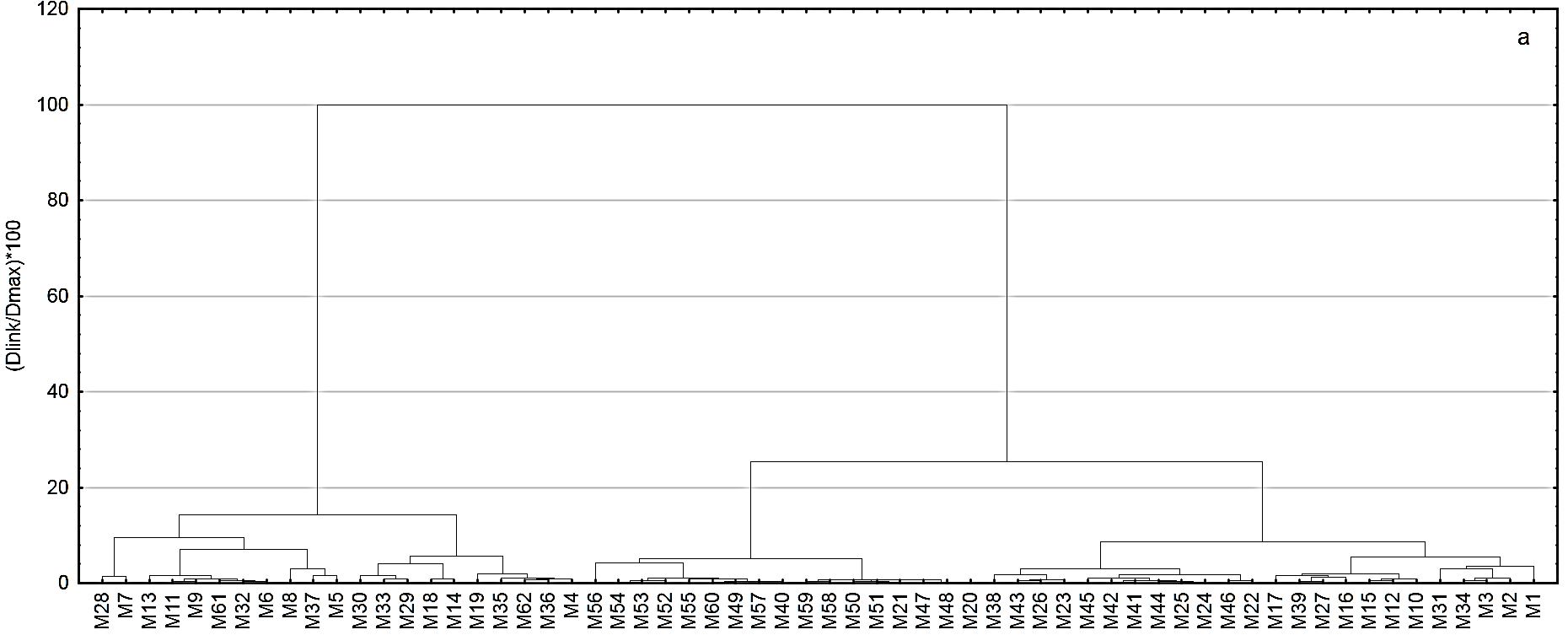


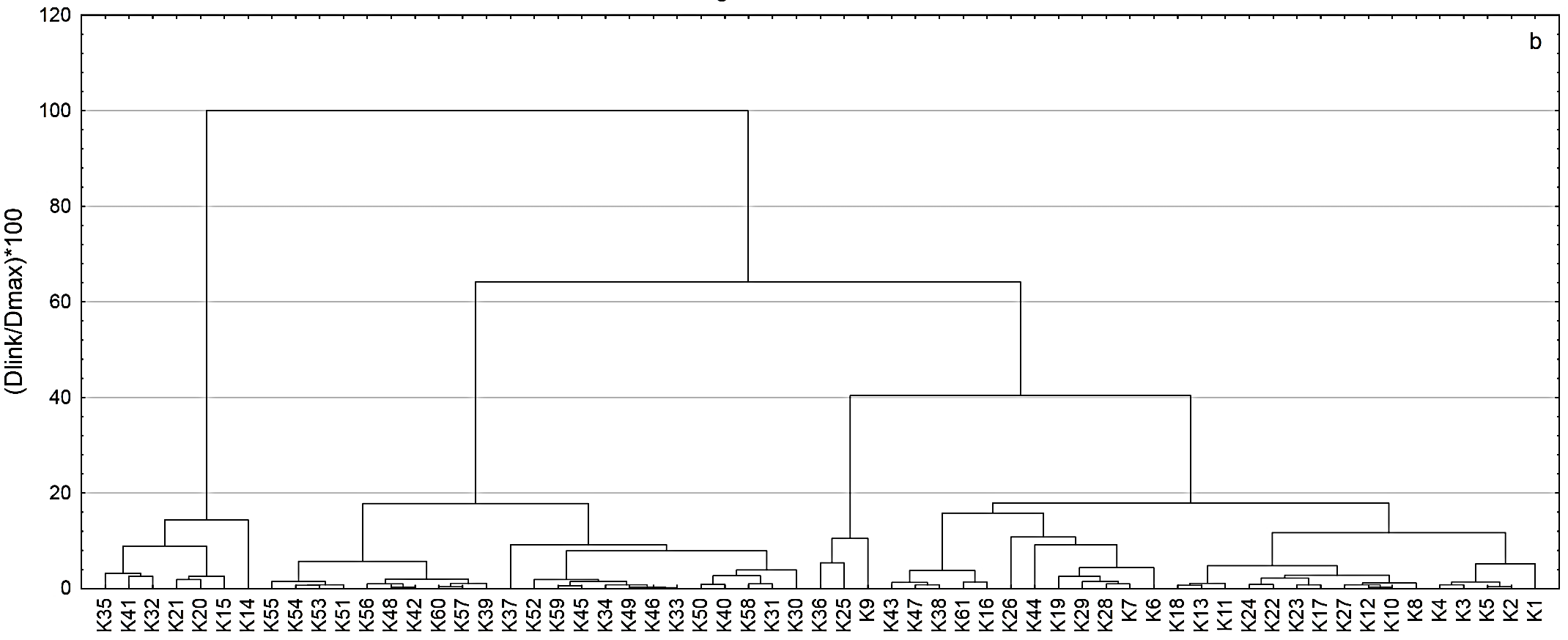


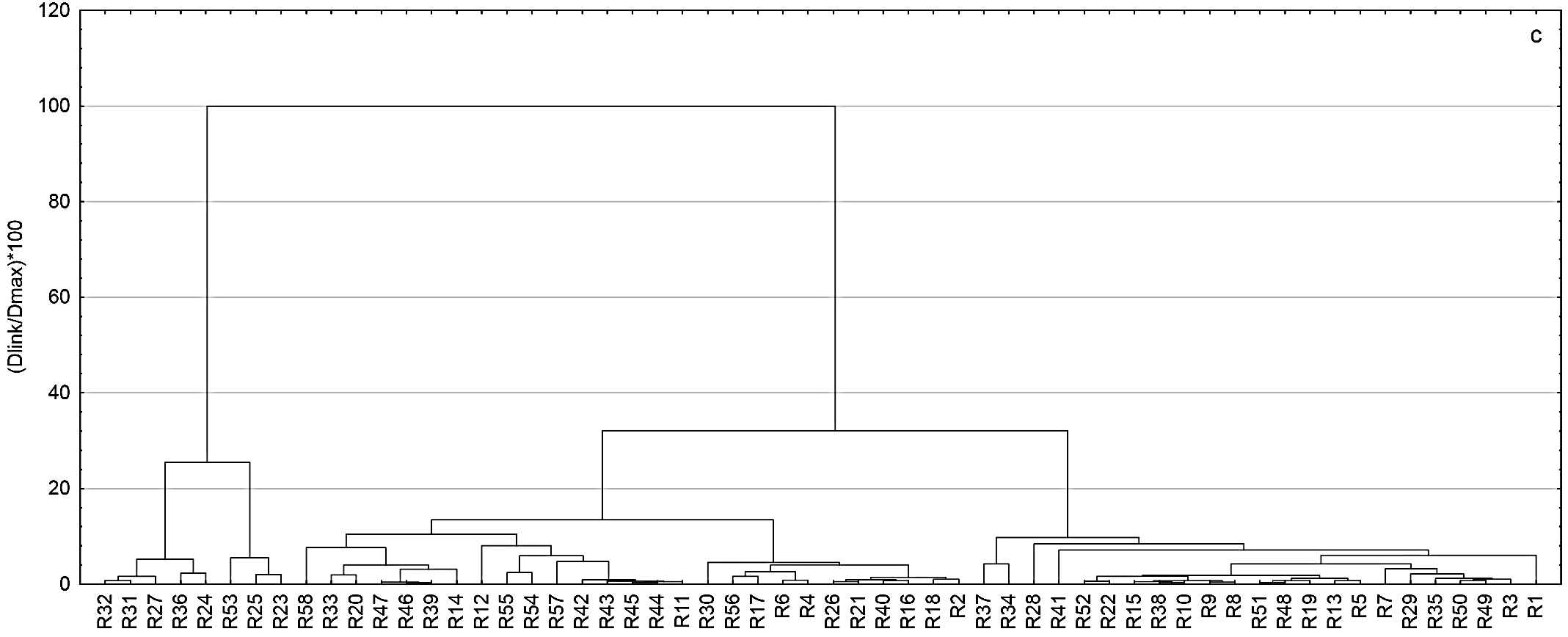


**Fig. 2** Tree diagrams obtained for non-mercury biased datasets from: a – Miedzianka Mt., b – Karczówka Mt., c – Rudki.
